# Development of a novel pyroptosis-related LncRNA signature with multiple significance in acute myeloid leukemia

**DOI:** 10.3389/fgene.2022.1029717

**Published:** 2023-01-04

**Authors:** Guangcai Zhong, Chong Guo, Yangli Shang, Zelong Cui, Minran Zhou, Mingshan Sun, Yue Fu, Lu Zhang, Huimin Feng, Chunyan Chen

**Affiliations:** ^1^ Department of Hematology, Qilu Hospital of Shandong University, Jinan, China; ^2^ The Second Hospital of Shandong University, Jinan, China

**Keywords:** acute myeloid leukemia, pyroptosis, lncRNA, prognosis, lasso-cox regression, tumor microenvironment

## Abstract

**Background:** Pyroptosis, a programmed cell death (PCD) with highly inflammatory form, has been recently found to be associated with the origin of hematopoietic malignancies. Long noncoding RNA (lncRNA) had emerged as an essential mediator to regulate gene expression and been involved in oncogenesis. However, the roles of pyroptosis-related lncRNA (PRlncRNA) in acute myeloid leukemia (AML) have not yet been completely clarified.

**Methods:** We collected AML datasets from public databases to obtain PRlncRNA associated with survival and constructed a PRlncRNA signature using Lasso-Cox regression analysis. Subsequently, we employed RT-PCR to confirm its expression difference and internal training to further verify its reliability. Next, AML patients were classified into two subgroups by the median risk score. Finally, the differences between two groups in immune infiltration, enrichment analysis and drug sensitivity were further explored.

**Results:** A PRlncRNA signature and an effective nomogram combined with clinicopathological variables to predict the prognosis of AML were constructed. The internal validations showed that the PRlncRNA risk score model was an accurate and productive indicator to predict the outcome of AML. Furthermore, this study indicated that higher inflammatory cell and immunosuppressive cells, and less sensitive to conventional chemotherapy drugs were highlighted in the high-risk group.

**Conclusion:** Through comprehensive analysis of PRlncRNA model, our study may offer a valuable basis for future researches in targeting pyroptosis and tumor microenvironment (TME) and provide new measures for prevention and treatment in AML.

## 1 Introduction

Acute myeloid leukemia (AML) is one of the severe and life threatening hematological malignant tumor arising from hematopoietic stem cells (HSCs) (Thomas and Majeti, 2017) with a high recurrence and mortality rate. About 10–40% of younger AML patients are primarily refractory to induction therapy, while a higher recurrence rate was observed in elderly patients (Döhner et al., 2015). After receiving allogeneic hematopoietic stem cell transplantation (allo-HSCT), up 50% of AML patients finally relapse (de Lima et al., 2014), and the 2-year survival rates are below 20% (Schmid et al., 2012; Schmid et al., 2018). Currently, guideline for the risk stratification of AML had included cytogenetic abnormalities, as well as gene mutations in *NPM1, CEBPA, FLT3,* and *KIT* (Dohner et al., 2017), improving the diagnosis and prognosis of AML patients. Although most AML patients could reach initial remission after induction chemotherapy, the long-term survival was still dismal. Accordingly, it is still urgently needed to find novel treatment strategies for improving the outcome of AML patients.

Pyroptosis, a highly inflammatory form of programmed cell death (PCD) (Miao et al., 2010), has garnered increasing attention as it relates to innate immunity and various diseases. Unlike apoptosis, pyroptosis was induced by the activation of gasderminsis through classical and non-classical pathways (Kayagaki et al., 2015; Ding et al., 2016; Liu et al., 2016; Rogers et al., 2017; Broz et al., 2020; Zhou et al., 2020), leading to cell swelling and plasma membrane rupture, and then triggering a strong inflammatory response (Chen et al., 2016). This process, however, could be either beneficial or detrimental to clearance of malignancies. Pyroptosis can inhibit the occurrence and development of tumors by mediating cell death and favourable immune response, while its hyper-inflammatory state could form a microenvironment conducive to tumor development and metastasis. During the inflammatory response, inflammasome activation exerts a critical role in maintaining multiple stages of hematopoietic homeostasis (Yang et al., 2021). Irreversible pyroptotic cell death may be caused by the activation of inflammasome in HSCs and thus leading to the origin of hematopoietic malignancies (Wei et al., 2022). An immunity and pyroptosis gene-pair signature performed stronger survival predictive efficacy than 10 existing signatures (Kong et al., 2022). The researches for the relationship between pyroptosis and tumor may provide some inspirations for clinical treatments. Recent several researches showed pyroptosis-related protein-coding genes could predict prognosis in AML (He et al., 2022; Liu et al., 2022; Pan et al., 2022; Shao et al., 2022; Zhou et al., 2022).

With a complex secondary and tertiary structure, long noncoding RNA (lncRNA) is RNA with over 200 nucleotides in length. Now, lncRNA has emerged as an essential mediator to participate in the regulation of gene expression such as chromatin modification, transcriptional regulation, and post-transcriptional regulation (Mercer et al., 2009) in almost all aspects of biology, particularly in tumorigenesis (Yang et al., 2014; Fang and Fullwood, 2016). Currently, accumulating researches have reported the effect of lncRNA as prognostic and diagnostic markers in leukemia (Garzon et al., 2014; Lei et al., 2018; Mer et al., 2018; Zhu et al., 2021). Moreover, several lncRNA recently were found to play roles in the pyroptosis pathway (He et al., 2020; Xu et al., 2020; Zhang et al., 2022). However, the relationship between pyroptosis-related lncRNA (PRlncRNA) and clinical prognosis in AML is still ambiguous.

In this study, we identified that PRlncRNA could effectively predict the outcome of AML and were associated with immunity and pro-inflammatory signaling. We first constructed a novel PRlncRNA prognostic model. Next, two risk groups were identified by the median risk score. The relationship between risk groups in immune infiltration, functional analysis and response to chemotherapy were further explored. Our study may facilitate an understanding of the mechanism underlying PRlncRNA in AML and provide precision therapies.

## 2 Materials and methods

### 2.1 Data collection

We downloaded the expression profiles of RNA-seq (n = 150) and clinicopathological information of AML tumorous tissue from the Cancer Genome Atlas (TCGA) website (https://portal.gdc.cancer.gov/), while the RNA-seq profiles of normal blood (*n* = 337) were obtained from UCSC XENA of Genotype-Tissue Expression (GTEx) (https://xenabrowser.net/datapages/) (Li et al., 2021). Then we normalized and processed the RNA-seq profiles of counts value from TCGA and GTEx with “limma” package (Mounir et al., 2019). From Genecards (https://www.genecards.org/), 176 pyroptosis-related genes (PRGs) (Supplementary Table S1) were finally retrieved.

### 2.2 Functional pathway enrichment of pyroptosis-related differentially expressed genes (DEGs)

According to the screening criteria of a false discovery rate (FDR) < 0.05 and |log2 fold change (FC)| > 0.25, 57 pyroptosis-related DEGs were obtained with the “limma” package in R (Ritchie et al., 2015). For better understanding of the functions of DEGs, functional enrichment analysis, including the GO terms and KEGG pathways were carried out with “clusterProfiler” and “ggplot2″ packages (Yu et al., 2012).

### 2.3 Construction and validation of the prognostic model

We evaluated the association between 57 pyroptosis-related DEGs and lncRNA by the pearson correlation coefficient. In total, 877 PRlncRNA were screened out following the criteria of absolute correlation coefficient >0.3 and a *p*-value < 0.001. To minimize the possible statistical bias, the samples were sifted when they lack overall survival (OS) value, of which 125 AML patients with complete clinical information were obtained. First, univariate Cox regression was applied to determine the lncRNA associated with prognosis. Subsequently, to prevent overfitting, least absolute shrinkage and selection operator (LASSO) regression was performed using the “glmnet” package. Finally, multivariate Cox regression was used to establish a four-lncRNA risk model of AML. We computed a risk score of each patient *via* the expression level of lncRNA and its regression coefficient. The formula was as follows: $Risk\;score=\overset{n}{\underset{i}{\sum }}Coefi\times A_{i}$ . AML patients were randomly assigned into high- and low-risk groups by median value of the risk score and a Kaplan–Meier survival curve was further employed with the “survival” package. Then we applied “survivalROC” package to plot the receiver operating characteristic (ROC) curve of the 1-, 3-, and 5-year for OS rates of AML. Patients of entire set were randomized into two internal test sets with “caret” package (Mahmoudian et al., 2021). Finally, the same above formula was used to further validate the reliability of the signature.

### 2.4 Construction of the nomogram and analysis of potential clinical relevance

We constructed a nomogram combining risk score and clinical factors (age and molecular risk) with the ‘rms’ package to predict the 1-, 2-, and 4-year survival of AML patients (Iasonos et al., 2008). Then calibration curves were plotted to assess the clinical utility of the nomogram. At first, we took 1-,3- and 5-year as the criteria for nomogram, however the calibration curve showed no score in 5-year. Thus, a 1-,3-, and 4-year setting was drawn back. For further exploration of the predictive signature, we compared the survival difference of risk groups and expression differences of predictive genes in clinicopathological subgroups.

### 2.5 Evaluation of immune status

First, Immune checkpoints activation was compared between risk groups using “ggpubr” package. In order to explore the association between risk scores and immune cells and functions, we employed the single-sample gene-set enrichment analysis (ssGSEA) score to quantify the enrichment levels of immune cells, related functions or pathways in different subgroups, using the “gene set variation analysis (GSVA)" package (Dalangood et al., 2020). Moreover, the proportions of 22 immune cells between the risk groups were explored by the CIBERSORT algorithm (Newman et al., 2015).

### 2.6 Gene set enrichment analysis (GSEA)

GSEA was carried out to determine the predominant genes enriched pathways with GSEA 4.1.0 (http://www.broad.mit.edu/gsea/). We applied “c2. cp.kegg.v7.5. symbols”, “c5. go.v7.5. symbols” and “h.all.v7.5. Symbols” to complete functional enrichment analysis. The threshold for statistical significance was considered as nominal *p* < 0.05 and FDR <0.25.

### 2.7 Drug sensitivity prediction

To evaluate the therapeutic value of signature in AML, the “pRRophetic” package (Geeleher et al., 2014) was applied to analyse the half inhibitory concentration (IC50) of typical chemotherapy drugs. Thereafter, we compare the IC50 values between two risk groups to search for potential drugs.

### 2.8 Human clinical specimens preparation

Samples of newly diagnosed AML and iron-deficiency anemia (IDA) were acquired from patients who were treated in the Qilu Hospital of Shandong University in Jinan, China. Ten pairs of bone marrow samples were included and IDA samples served as control.

### 2.9 RNA extraction and real-time quantitative PCR (RT-qPCR)

Total RNA was extracted using the Trizol method as previously reported (Invitrogen, Carlsbad, CA, United States). Complementary DNA (cDNA) was synthesized using Evo M-MLV RT Mix Kit with gDNA Clean for qPCR (Accurate Biology, Human, China). Four lncRNA gene expressions were verified by PCR using SYBR^®^ Green Premix Pro Taq HS qPCR Kit (Accurate Biology, Human, China) with GAPDH as a control. The primer sequences of four lncRNA were shown in Supplementary Table S2.

### 2.10 Statistical analysis

Results of the following analysis were performed with the R software (Version 4.1.0). Comparison of OS between risk groups was conducted by Kaplan-Meier analysis as well as log-rank tests. The time-dependent ROC curve and the area under curve (AUC) were carried out with the “timeROC” package. For continuous variables, Student’s t-test and Wilcox test were performed to test the differences between two groups while One-way ANOVA or Kruskal–Wallis test was used in three or more groups. Chi-square tests were employed in categorical variables. The optimal cut-off value was 0.876 between two risk groups determined by “survminer” package. P-values are two-sided in all statistical, and *p* < 0.05 was considered as statistically significant results.

## 3 Results

### 3.1 Identification and enrichment analysis of pyroptosis-related DEGs

**FIGURE 1:**
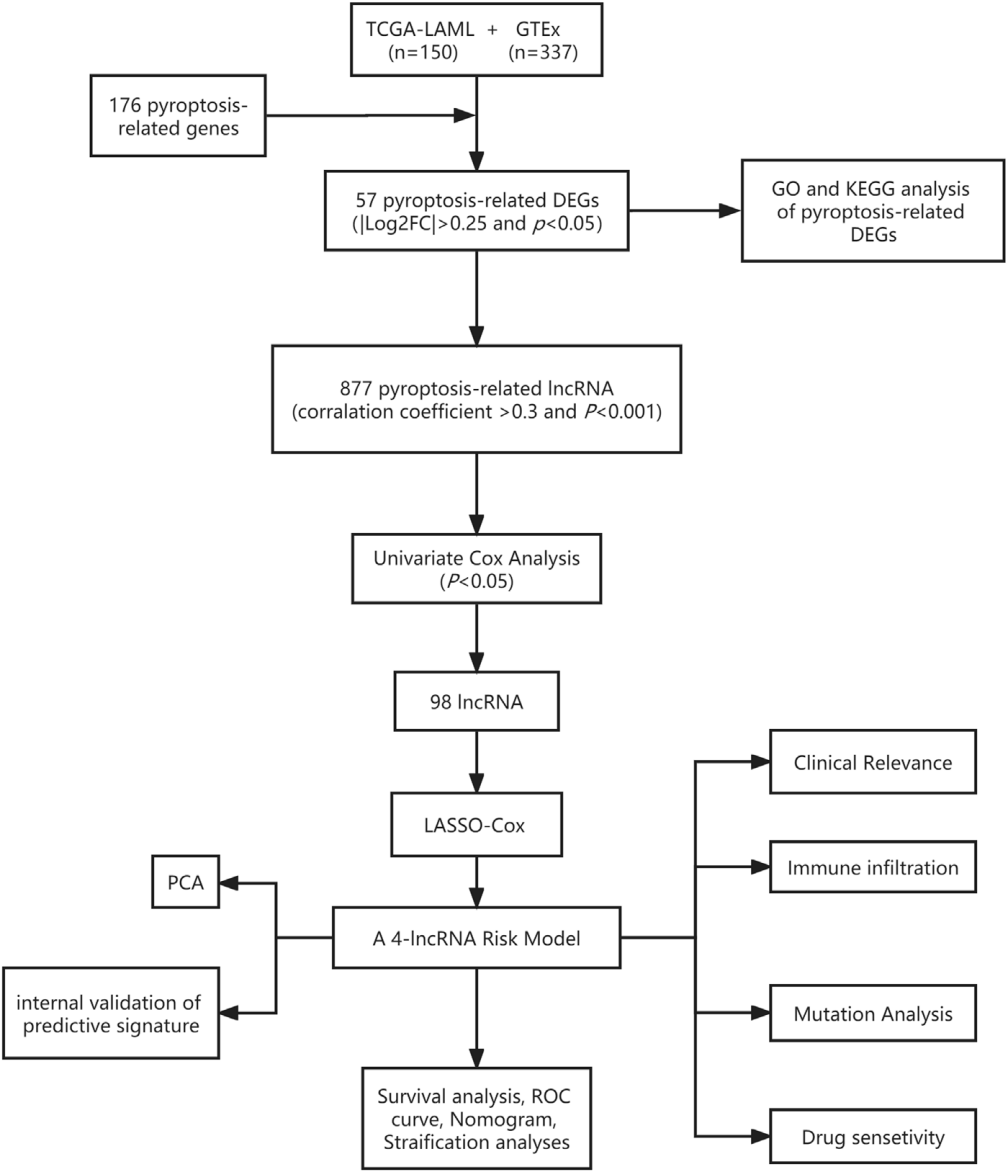
Detailed flow chart of our research.

**FIGURE 2:**
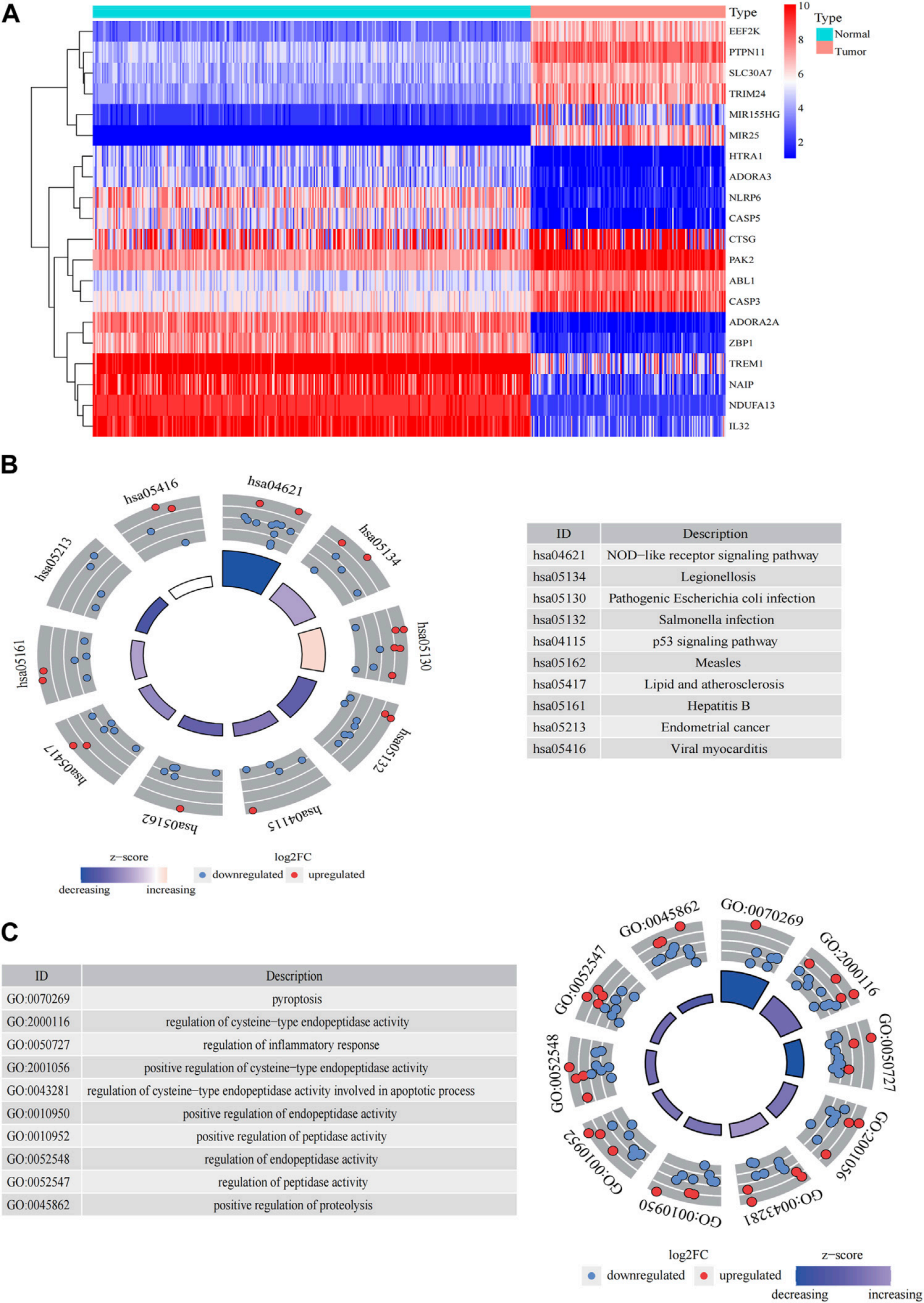
Significantly enriched GO terms and KEGG pathways of pyroptosis-related DEGs between AML and the healthy control. **(A)** Heatmap of top 10 pyroptosis-related DEGs of upregulated and downregulated genes. Enrichment analysis of pyroptosis-related DEGs of KEGG pathway **(B)** and GO terms **(C)**.

The general workflow (Figure 1) describes the whole screening and analysis processes. From TCGA and GTEx matrix, we obtained 150 tumor samples and 337 normal samples. According to the expressions of PRGs and DEGs (|Log2FC| > 0.25 and *p* < 0.05), 57 pyroptosis-related DEGs including 35 downregulated genes and 22 upregulated genes were obtained (Supplementary Figure S1A). The expression level of the top 10 upregulated and downregulated pyroptosis-related DEGs were characterized (Figure 2A). Then KEGG and GO analysis were performed. Pyroptosis-related DEGs were mainly enriched in the NOD−like receptor signalling pathway, Legionellosis, Pathogenic Escherichia coli infection, *Salmonella* infection, p53 signalling pathway, Measles, Lipid and atherosclerosis, Hepatitis B, Endometrial cancer and Viral myocarditis according to KEGG pathway analysis (Figure 2B; Supplementary Table S3). Furthermore, pyroptosis, regulation of cysteine−type endopeptidase activity, regulation of inflammatory response and positive regulation of proteolysis were highlighted in GO enrichment (Figure 2C; Supplementary Table S4).

### 3.2 Establishment and validation the pyroptosis-related prognostic model

**FIGURE 3:**
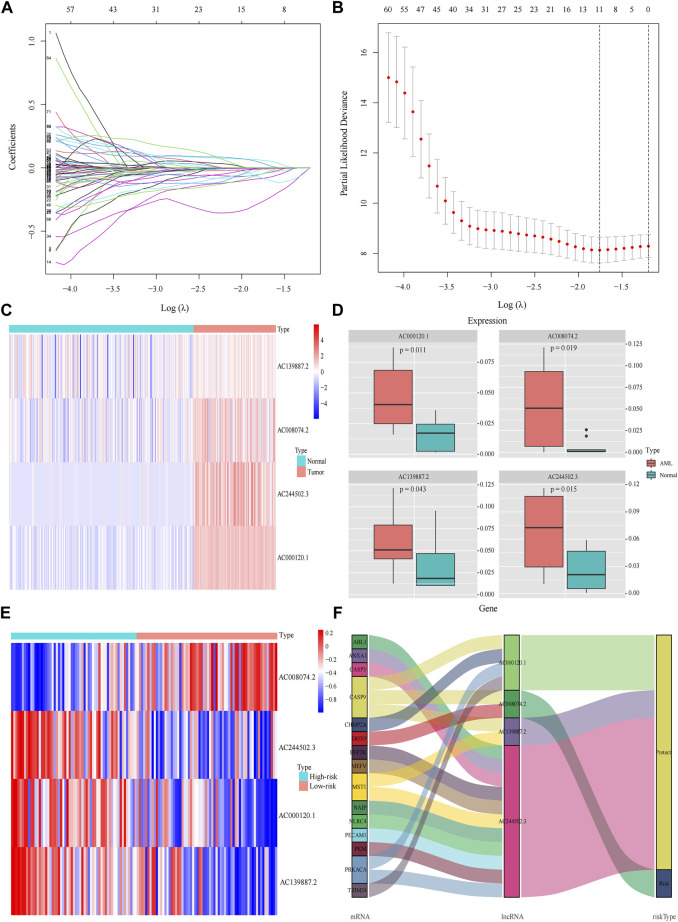
Construction the pyroptosis-related prognostic model by DEGs. **(A,B)** Least absolute shrinkage and selection operator (LASSO) regression analysis with ten-fold cross validation to determine the lambda number. **(C)** The expression levels of four pyroptosis-related lncRNA in AML and healthy control (Wilcoxon tests). **(D)** RT-PCR-detected RNA expression of four genes in our own samples, 10 AML *de novo*, and 10 IDA as normal control samples. **(E)** The expression levels of four pyroptosis-related lncRNA in different risk groups. **(F)** Sankey diagram of prognostic pyroptosis-based lncRNA.

**FIGURE 4:**
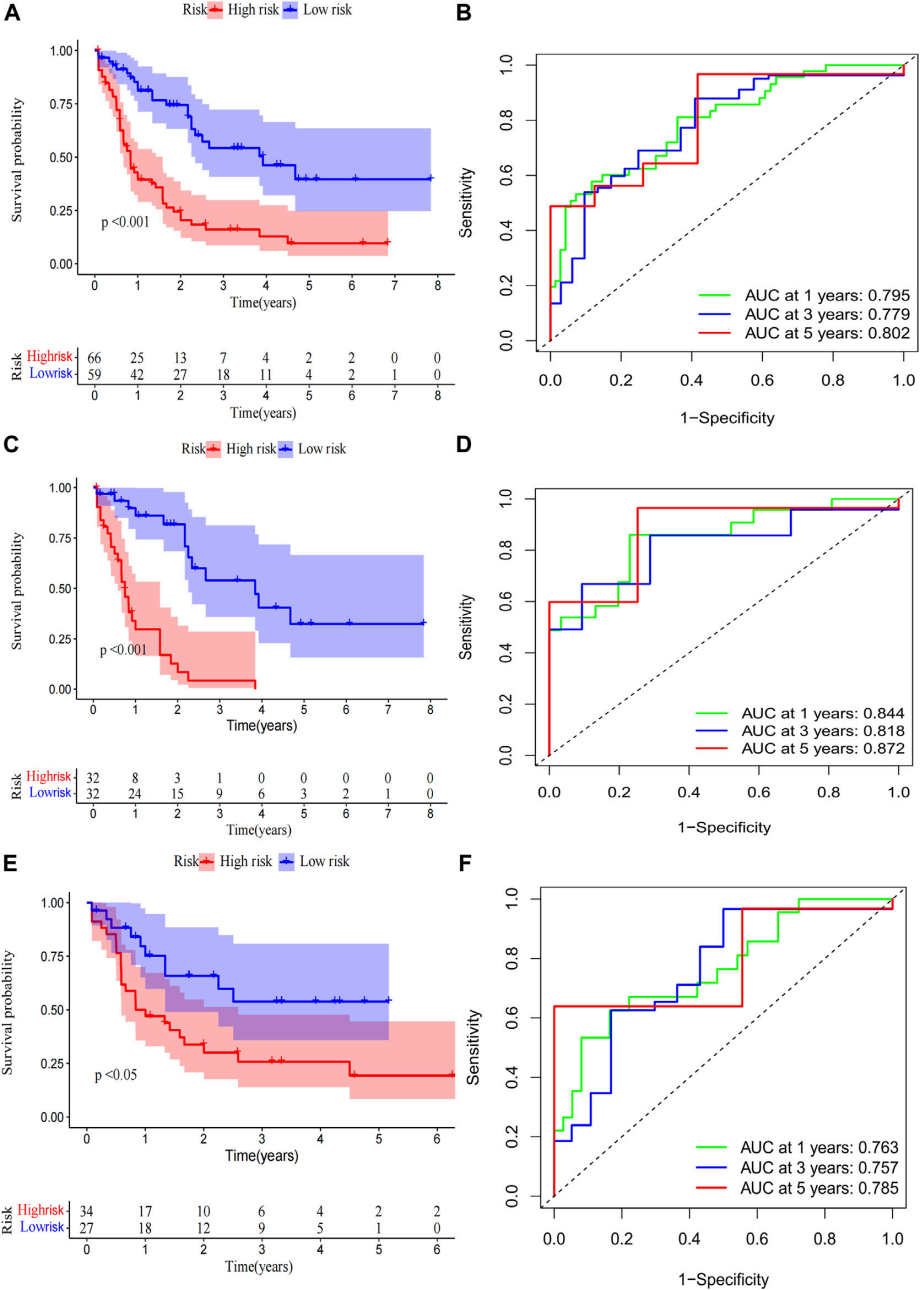
Risk score of the pyroptosis-related prognostic signature for overall survival (OS). Kaplan-Meier survival curve of high- and low-risk patients and ROC curve and AUCs at 1-year, 3-year and 5-year survival in the entire cohort **(A,B)**, the first internal cohort **(C,D)** and the second internal cohort **(E,F)**.

A total of 98 PRlncRNA correlated with OS were identified by univariate Cox regression (Supplementary Figure 1B). Then, we used the LASSO-Cox regression to reduce overfitting (Figures 3A,B). Finally, a pyroptosis-related prognostic risk model composed of four lncRNA (*AC244502.3*, *AC000120.1*, *AC139887.2*, *AC008074.2*) was established by multivariate Cox regression. Higher expression level of four PRlncRNA were observed in tumor tissues (Figure 3C). Their higher expressions in AML patients were validated according to RT-qPCR (Figure 3D). *AC244502.3*, *AC000120.1* and *AC139887.2* were up-regulated in low-risk group as protective factors in this prognostic model, while *AC008074.2* was a risk factor (Figures 3E,F). The risk score was as follows: risk score = *AC008074.2* expression * 0.751252629082265 + *AC139887.2* expression * (-1.43187457646711) + *AC000120.1* expression * (-1.49861878505506) + *AC244502.3* expression * (-0.278256123715056). We further visualized the correlation between lncRNA and mRNA. The co-expression network including 19 pairs mRNA-lncRNA was characterized in Supplementary Figure 2A (|R2 | > 0.3 and *p* < 0.001). Two risk groups were randomised by the median value of the risk score. The patients of high-risk group were observed significantly shorter OS than those in low-risk group (Figure 4A). ROC curve was applied to assess the reliability of our prognostic signature. The AUC value was 0.795 at 1 year, 0.779 at 3 years, and 0.802 at 5 years which indicated excellent stability (Figure 4B). To determine the prediction ability of the model, we stratified entire dataset into two groups. Similar results were observed in two internal cohorts. The high-risk group showed lower OS rate and good evaluated prognostic power was also observed in validation cohorts according to the ROC curves (Figures 4C–F). The 1-,3-, and 4-year ROC curves for entire cohort and two internal cohorts were shown in Supplementary Figure S2B–D. Patients’ baseline characteristics of the entire and two validation cohorts are outlined in Table 1.

**TABLE 1:** The clinical characteristics of patients in different cohorts.

	Entire TCGA dataset (n = 125)	Validation cohorts		
		First cohort (n = 61)	second cohort (n = 64)	*p*-value
Age (Mean ± SD)	54.1 ± 1.4	53.4 ± 2.0	54.7 ± 2.1	0.514
Gender, n (%)				0.063
Female	55 (44.0%)	29 (47.5%)	41 (64.1%)	
Male	70 (56.0%)	32 (52.5%)	23 (35.9%)	
FAB, n (%)				0.930
Non-M3	113 (90.4%)	55 (90.2%)	55 (90.2%)	
M3	12 (9.6%)	6 (9.8%)	6 (9.4%)	
WBC, ×10^9/L (Mean ± SD)	30.5 ± 3.2	33.6 ± 5.1	27.7 ± 4.0	0.516
HB, g/L (Mean ± SD)	9.6 ± 0.1	9.7 ± 0.2	9.5 ± 0.2	0.700
PLT, ×10^9/L (Mean ± SD)	66.6 ± 5.0	68.8 ± 6.5	64.5 ± 7.7	0.261
PB_blast_cell (Mean ± SD)	66.2 ± 2.0	65.9 ± 3.1	66.5 ± 2.5	0.906
BM_blast_cell (Mean ± SD)	36.9 ± 2.7	36.4 ± 4.0	37.4 ± 3.8	0.943
^a^RISKCyto, n (%)				0.805
Favorable	28 (22.4%)	14 (23.0%)	14 (21.9%)	
Intermediate	71 (56.8%)	33 (54.1%)	38 (59.4%)	
Poor	26 (20.8%)	14 (23.0%)	12 (18.8%)	
^b^RISKMole, n (%)				0.906
Favorable	29 (23.2%)	15 (24.6%)	14 (21.9%)	
Intermediate	68 (54.4%)	32 (52.5%)	36 (56.3%)	
Poor	28 (22.4%)	14 (23%)	14 (21.9%)	
Riskscore (Mean ± SD)	1.9 ± 0.2	2.1 ± 3.5	1.6 ± 0.2	0.374

^a^ Cytogenetic risk.

^b^ Molecular risk.

### 3.3 Construction of nomogram and analysis of the potential clinical relevance of the prognostic signature

**FIGURE 5:**
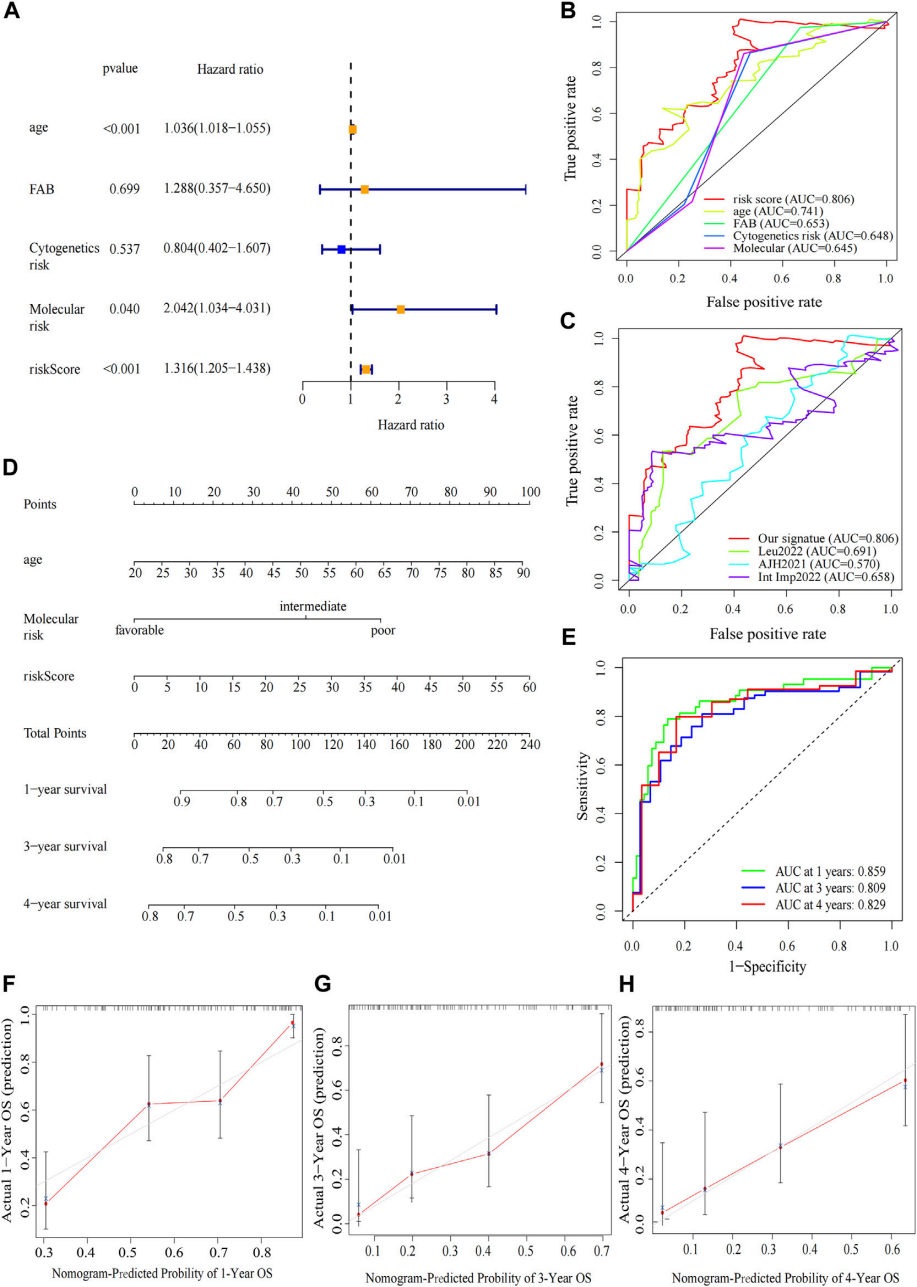
Construction and verification of the nomogram. **(A)** Forest map of multivariate Cox regression analysis of the risk scores and clinical parameters. **(B)** The ROC curve of multivariate Cox regression variables. **(C)** Prognostic ROC AUC comparison of Our signature and other three signatures. **(D)** The nomogram of 1-year, 3-year or 4-year OS of AML patients based on risk score, age and molecular risk. **(E)** ROC curves and AUCs for the nomogram. **(F–H)** The calibration curves test consistency between the actual OS rates and the predicted survival rates at 1, 3 and 4 years.

The clinical relevance of our signature was further explored. We performed univariate Cox regression analysis incorporating eleven variables that were easily accessible (Supplementary Figure 2E). Besides risk score, molecular risk and age were shown to be independent risk factors for OS and then were enrolled to multivariate Cox regression (Figure 5A). The risk score demonstrated a higher predictive ability than that of clinicopathological variables in AML (Figure 5B) and other previously reported prognostic signatures [Leu 2022 (Kong et al., 2022), AJH 2021 (Chen et al., 2021), Int Imp 2022 (Shao et al., 2022)] (iAUC-Our signature: 0.806, iAUC-Leu2022: 0.691, iAUC-AJH2021: 0.570, and iAUC-IntImp2022: 0.658; all the *p*-values in comparisons between Our signature and the above three models were <0.001). Based on multivariate Cox regression, we developed a novel nomogram, aiming to optimize the predictive accuracy of the risk model (Figure 5D). The ROC curve exhibited a reliable predictive efficacy (Figure 5E). The 1-,2-, and 4-year calibration curves of our constructed nomogram yielded good agreement between prediction and observation (Figures Figure5F–H). The clinical characteristics of two risk groups were further compared. The heatmap showed that the distribution of FAB type varied significantly in risk groups while age, gender, molecular risk, cytogenetic risk and blood cell counts were no statistically differences (Supplementary Figure S3). To assess the predictive ability of the predictive signature in different clinicopathological subgroups, we separated AML patients into groups by age, sex, FAB, molecular risk and cytogenetic risk. For each clinical subgroups, the patients of high-risk group had worse OS than those of low-risk group (Supplementary Figure S4). We further explored association between the four predictive genes and clinical variables (Supplementary Figure S5).

### 3.4 Enrichment analysis of the prognostic model

**FIGURE 6:**
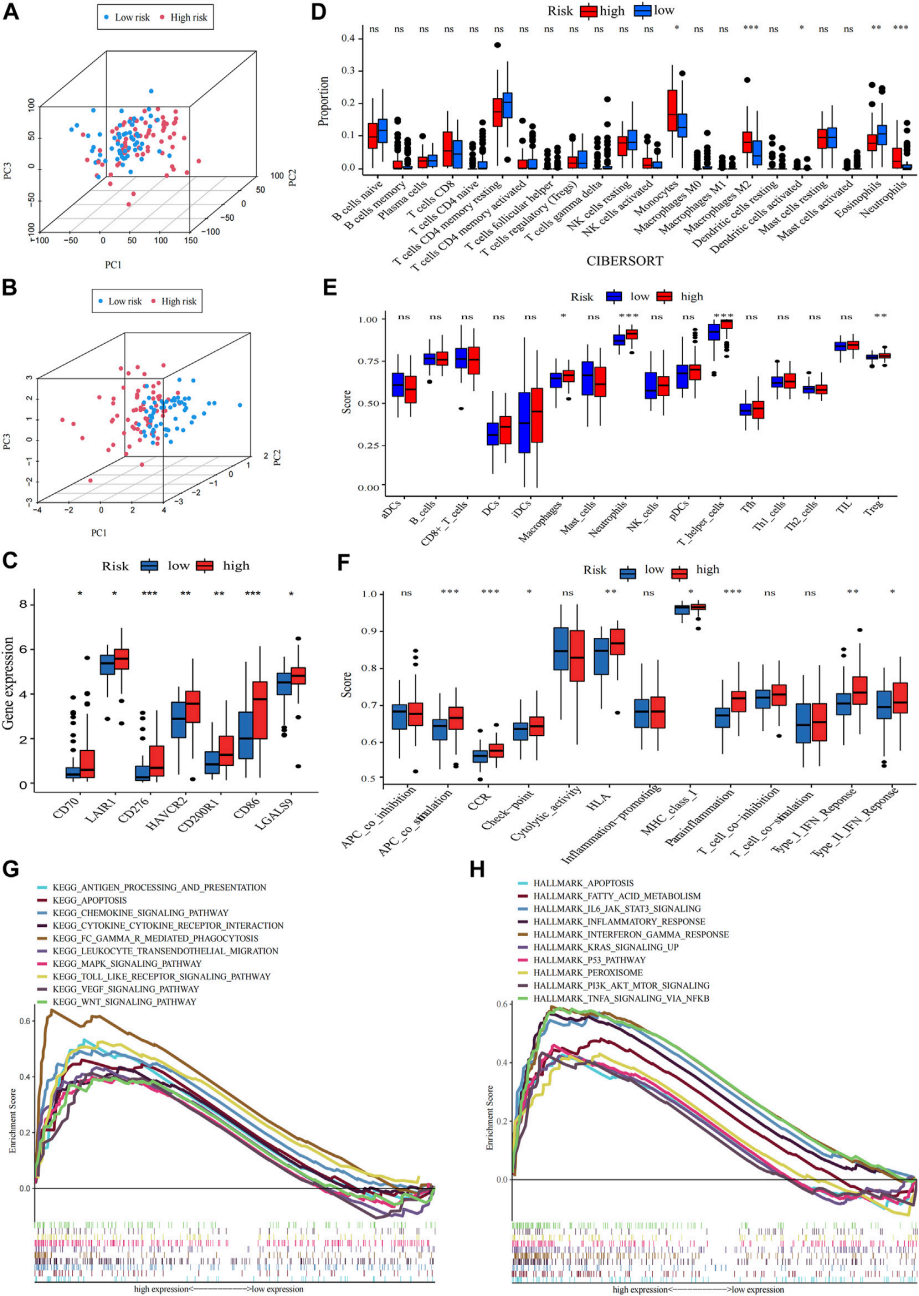
Analysis of differences in immunological characteristics and functional enrichment of AML in different risk groups. **(A,B)** PCA analysis of the expression patterns of grouped samples using all genes and prognostic signature. **(C)** Differential expression of immune checkpoint genes in low- and high-risk group (Wilcox test). **(D)** The proportion of every type of TME infiltrating cells between the two risk groups analyzed, respectively, by CIBERSORT. **(E,F)** Differences between immune cell infiltration and immune-related functions in two subtypes, with ssGSEA algorithm (Wilcoxon test, *p < 0.05; **p < 0.01; ***p < 0.001; ns, non-significant). **(G,H)** The high-risk group enriched gene sets of GSEA-based KEGG and Hallmark analysis.

**FIGURE 7:**
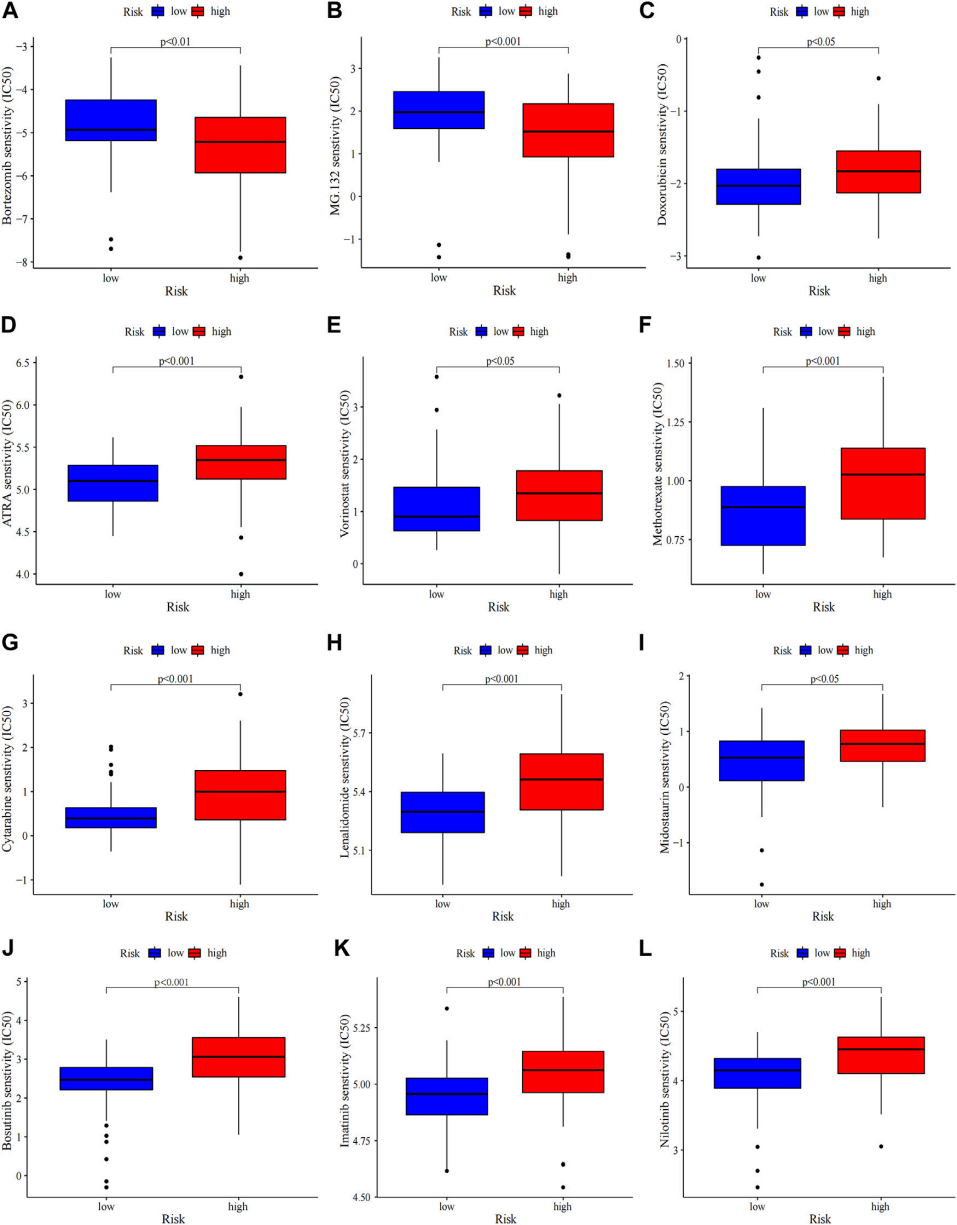
Chemosensitivity prediction. Higher drug sensitivity in high-risk group **(A–C)** and low-risk group **(D–L)** (Wilcoxon tests). Lower half inhibitory centration (IC50) means better drug sensitivity.

Principal component analysis (PCA) indicated that AML patients could not be well separated into high- and low-risk groups by all genes (Figure 6A) but the shortfalls could be compensated by our signature (Figure 6B). The genes of immune checkpoint such as CD70, LAIR1, CD276, HAVCR2, CD200R1, CD86 and LGALS9, were higher expressed in high-risk group, demonstrated that high-risk group may profit from immune checkpoint inhibitors (Figure 6C). Subsequent analysis of the tumor microenvironment was carried out. Inflammatory and immunosuppressive cells were abundant in high-risk group, including the presence of Monocytes, Neutrophils along with M2 macrophages, thus conferring a significant survival disadvantage (Figure 6D). Then, we investigated the differences in immune signatures between low- and high-risk groups. Neutrophils, tregs, chemokine receptor (CCR), check−point, type-I and type-II IFN response and parainflammation were observed with higher expression level in high-risk group, indicating that high-risk group was correlated with the inflammatory response and tumor immune escape (Figures 6E,F). To assess the potential biological processes between two groups, enrichment analysis of GO, KEGG and hallmarker were performed (Figures 7G,H; Supplementary Figure S6A; Supplementary Tables S5–7). High-risk patients were closely related to myeloid leukocyte activation, inflammatory response such as Chemokine, Cytokine-cytokine receptor interaction, Interleukin-6 production, Leukocyte transendothelial migration, Toll like receptor, Apoptosis and Interferon gamma, and tumor-related signaling pathways such as IL6-JAK-STAT3, MAPK, VEGF, Wnt, PI3K-AKT-mTOR, KRAS, NF-κB and P53. The inflammasome-related genes (Liu et al., 2016) exhibited a high expression level in the high-risk group (Supplementary Figure S6B).

### 3.5 Mutation profile and chemotherapeutics of the prognostic model

Gene mutations in AML could modify the disease process and subsequently influence the outcomes (Daver et al., 2019). Therefore, gene mutation status was analysed, among which *FLT3* had the highest mutation rate (Supplementary Figure S7A). There were no statistically differences in tumor mutation burden (TMB) between risk groups and survival rates between the TMB groups (Supplementary Figure S7B, C). The high-TMB group was observed poor prognosis in subgroup analysis stratified by risk groups (Supplementary Figure S7D). Then the 20 highest point mutations were further compared (Supplementary Figure S7E). Higher frequencies of *TP53* mutations (low vs. high: 0–11%) was found in the high-risk group (Supplementary Figure S7F). Chemotherapy is still the mainstream treatment for AML. We analysed the association between risk groups and the sensitivity to typical chemotherapeutic agents in AML (Figure 7). The low-risk group showed more sensitivity to the conventional chemotherapeutics including Doxorubicin, Cytarabine, Methotrexate, Etoposide, Midostaurin, Lenalidomide, ATRA and HDAC inhibitor Vorinostat, while the high-risk group was less resistant to protease inhibitor such as bortezomib and MG.132. Our risk signature may become a potential indicator of drug sensitivity.

## 4 Discussion

AML is an aggressive hematologic malignancy with complex and dysregulated microenvironment that, in part, promotes leukemogenesis (Teague and Kline, 2013). During the leukemic transition, AML blasts modified the immune microenvironment to evade immune surveillance. Accumulating evidences indicated that chronic inflammation in tumor microenvironment (TME) played a major role in immune invasion, thus contributing to tumorigenesis (Grivennikov et al., 2010; Shin and Brusselle, 2014; Lu et al., 2021). In some studies of pyroptosis in AML, TME was found remarkably different between risk groups (He et al., 2022; Shao et al., 2022; Zhou et al., 2022). Pyroptosis, an inflammation-dependent form of PCD, was proved to participate in tumor growth and chemosensitivity (Xia et al., 2019), while its mechanisms are complicated. For instance, pyroptosis serves as a tumor inhibitor in hepatocellular and nasopharyngeal carcinoma (Zaki et al., 2010; Chen et al., 2012) and a two-edged sword of promotor and inhibitor in cervical cancer (Xia et al., 2019). A growing body of evidences indicated that lncRNA participated in multiple biological processes of various tumors *via* pyroptosis-related pathways. ADAMTS9-AS2 inhibited gastric cancer progression and promoted cisplatin chemosensitivity by regulating the pyroptosis process (Ren et al., 2020). Moreover, the activation of MEG3 enhanced Cisplatin-induced pyroptosis in triple-negative breast cancer (Yan et al., 2021). In liver cancer, SNHG7 suppressed the NLRP3-related pyroptosis pathway (Chen et al., 2020). The effect of PRlncRNA in AML has not been yet clarified. Therefore, a PRlncRNA signature was established to explore its possible clinical relevance with AML.

In this study, 57 pyroptosis-related DEGs were obtained which were highly related to pyroptosis, inflammatory, p53 signaling pathway and positive regulation of endopeptidase activity based on KEGG and GO analysis. The genes of positive regulation of endopeptidase activity were related in numerous cellular processes, including apoptosis, DNA damage repair, or cell cycle progression (Jorgensen et al., 2006) and mediated PCD(Yamada et al., 2020). Next, we used Lasso-Cox regression analysis to identify a risk model of four-PRlncRNA (*AC244502.3, AC000120.1, AC139887.2 and AC008074.2*). Then, the signature was validated in two internal cohorts. The ROC curves of whole dataset and two internal training sets demonstrated high accuracy of the clinical prognostic model. Our signature exhibited greater predictive ability than clinicopathological variables such as molecular risk and cytogenetic risk. In our study, *AC244502.3*, *AC000120.1* and *AC139887.2* were observed as potentially protective lncRNA with higher expression in low-risk group, while *AC008074.2* was potentially dangerous. *AC000120.1* had been found to associated with the prognosis of Bladder Cancer (Cui et al., 2021). The functions of the three remaining lncRNA have not been studied and reported specifically yet. Thus, more researches of lncRNA are needed to be conducted.

The recruitment of inflammatory cells, cytokines and chemokines results in the inflammatory TME which contributes to metastasis and invasion of tumor cells, modulates the anti-tumor immune response and influences the sensitivity to chemotherapeutic drugs. Previous studies suggested the high-risk group of pyroptosis-related signature was enriched in immunosuppressive cells and had lower drug sensitivity to classical chemotherapy (Pan et al., 2022; Shao et al., 2022; Zhou et al., 2022). In pediatric patients with AML, PRGs were found to predict recurrence (He et al., 2022). To probe the underlying mechanisms further of our signature, the TME and enrichment analysis between risk subgroups were investigated. In this study, the high-risk group demonstrated higher inflammatory response mediated by IL-6-related signaling pathways and lower immune response regulated by immunosuppressive cells such as M2 macrophages, Treg cells and so on. Recent study indicated that M2 macrophages could induce immune suppression (Ruffell and Coussens, 2015) and were associated with the worse survival status (Italiani and Boraschi, 2014). Monocytes preferentially differentiated into immunosuppressive tumor-associated macrophages (TAMs) in solid tumor (Richards et al., 2013), which would suppress immune response. Higher level of IL-6 was considered to be related to bone marrow failure (Zhang et al., 2020) and the high risk of relapse for AML (Stevens et al., 2017). In TME, IL-6 could drive tumor cells proliferation, invasiveness, and metastasis through activating JAK/STAT3 signaling pathway which could in return promote IL-6 transcription (Chang et al., 2014). Meanwhile, NF-κB was identified as a key transcription factor that drove the IL-6 signaling (Yoon et al., 2012; McFarland et al., 2013). Receptor activator of nuclear NF-κB was associated with dismal disease course and chemoresistance in AML (Clar et al., 2021). The signaling pathway of JAK/STAT3 and NF-κB have been universally recognized as the bridge linking tumor and inflammation. As is well known, the inflammation induced by bacterial and viral infections could increase the cancer risk. Intrinsic inflammatory response could be triggered by tumors which may build up a protumor microenvironment (Tye et al., 2012). Additionally, upregulated procancer pathways (Wnt, p53, PI3K-AKT-mTOR, VEGF, and KRAS) were seen more frequently in high-risk group. This complicated cancer-immune crosstalk in the TME finally facilitate tumor cell growth and survival, with decreased antitumor immunity. Our research also showed that the low-risk group was less resistant to the conventional chemotherapy drugs, while high-risk patients may profit from proteasome inhibitor. Bortezomib may exert anti-inflammatory effects by inhibiting the expression of NF-κB, IL-6 and TNF-α. (Teague and Kline, 2013). Summarizing all findings, we constructed two groups, of which high-risk group had lower immune response, more inflammatory cell infiltration, and lower sensitivity to classical chemotherapy; together these characters resulted in a significant reduction in the overall survival.

In this study, based on four PRlncRNA, we constructed a prognostic risk score model for AML patients which demonstrated a reliable predictive validity. Moreover, the risk score model increased the knowledge of the mechanisms of PRlncRNA in AML and had the potential to provide more precisely targeted interventions. There are still some shortcomings that must be noted. First, external validation was not conducted due to the lack of expression profiles of lncRNA in other databases. Second, although with the validation of differential expression between normal and tumor samples, our study mainly based on public databases. Further experiments *in vivo* and *in vitro* should be needed to explore the underlying mechanisms of PRlncRNA in AML.

## Data Availability

The datasets presented in this study can be found in online repositories. The names of the repository/repositories and accession number(s) can be found in the article/Supplementary Material.

## Abbreviations

LncRNA, Long noncoding RNA; PRlncRNA, pyroptosis-related lncRNA; AML, Acute myeloid leukemia; allo-HSCT, allogeneic hematopoietic stem cell transplantation; HSCs, hematopoietic cells; TCGA, The Cancer Genome Atlas; GTEx, Genotype-Tissue Expression; DEGs, Differentially Expressed Genes; FDR, false discovery rate; OS, overall survival; LASSO, least absolute shrinkage and selection operator; ROC, receiver operating characteristic; ssGSEA, single-sample gene-set enrichment analysis; GSVA, gene set variation analysis; IC50, the half inhibitory centration; RT-qPCR, Real-Time Quantitative PCR; IDA, iron-deficiency anemia; cDNA, complementary DNA; AUC, the area under curves; PCA, principal component analysis; CCR, chemokine receptor; TMB, tumor mutation burden; TME, tumor microenvironment; PCD, programmed cell death.
