# Supplementary material for: Development of a novel pyroptosis-related LncRNA signature with multiple significance in acute myeloid leukemia
Source: Front Genet. 2023 Jan 4;13:1029717. doi: 10.3389/fgene.2022.1029717 (PMC9845279; doi:10.3389/fgene.2022.1029717)
Supplement: Supplementary file 1 [file DataSheet1.docx]

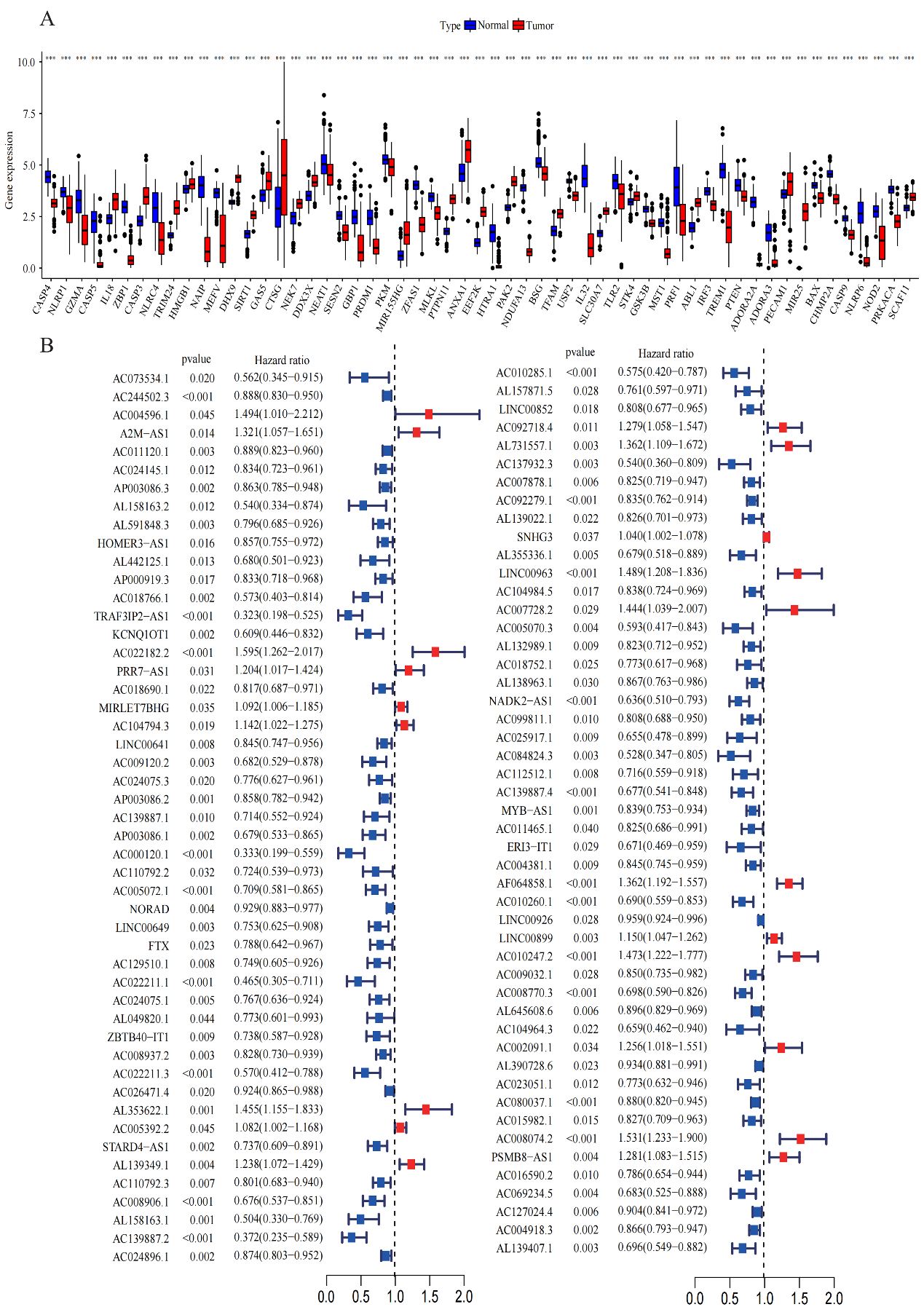


**Supplementary Figure 1** **The pyroptosis-related differentially expressed genes and prognosis related lncRNA.** (A) 57 pyroptosis-related differentially expressed genes in AML and control (Wilcoxon test, *p < 0.05; **p < 0.01; ***p < 0.001; ns, non-significant). (B) Forest map of univariate Cox regression analysis of PRlncRNA.


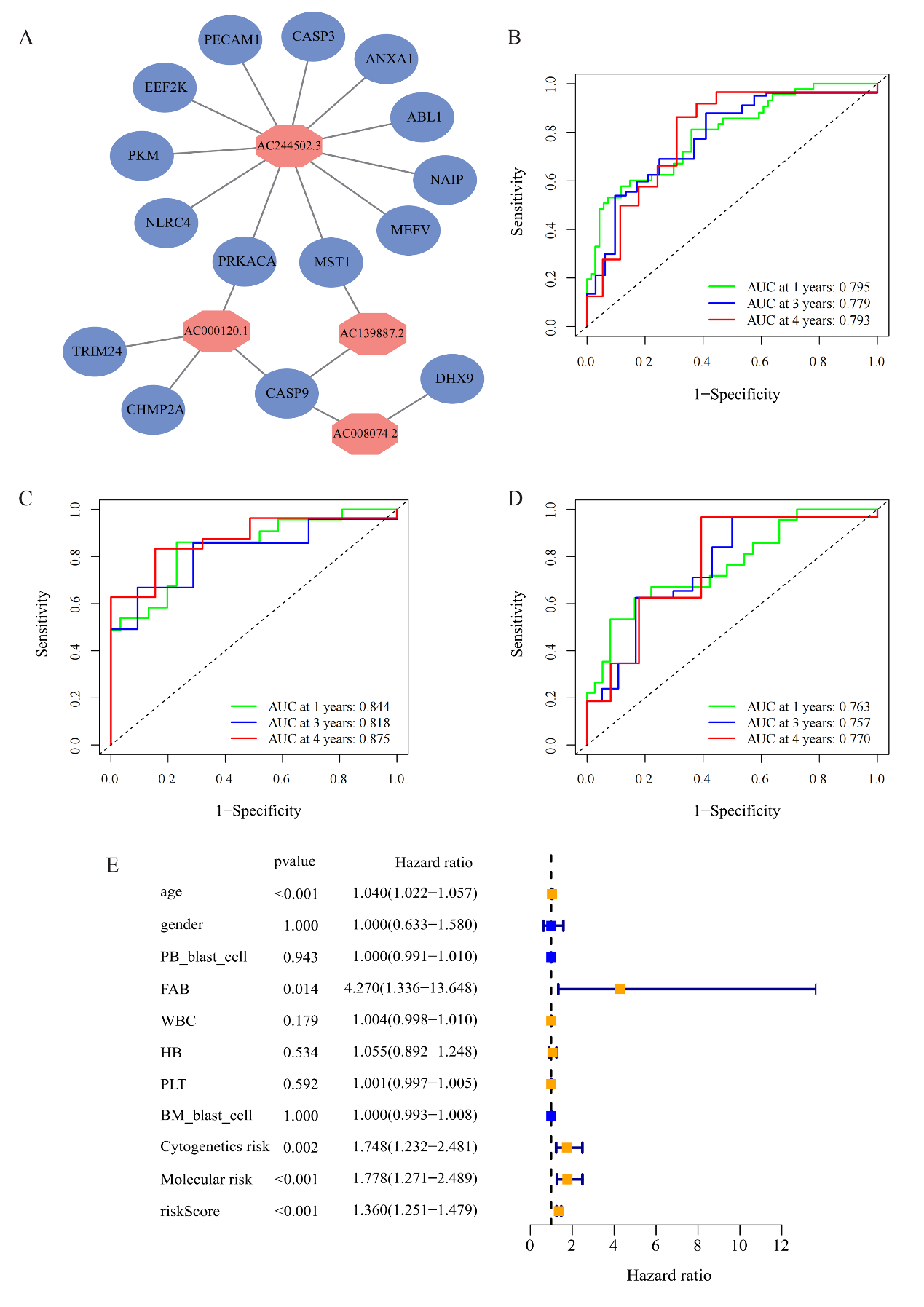


**Supplementary Figure 2** **The validation of risk score and analysis of risk factors associated with survival.** (A) The lncRNA-mRNA co-expression network. ROC curves and AUCs at 1-year, 3-years and 4-years survival in the entire cohort (B), the first internal cohort (C), and the second internal cohort (D). (E) Univariate Cox regression analysis of the risk scores and clinical parameters.


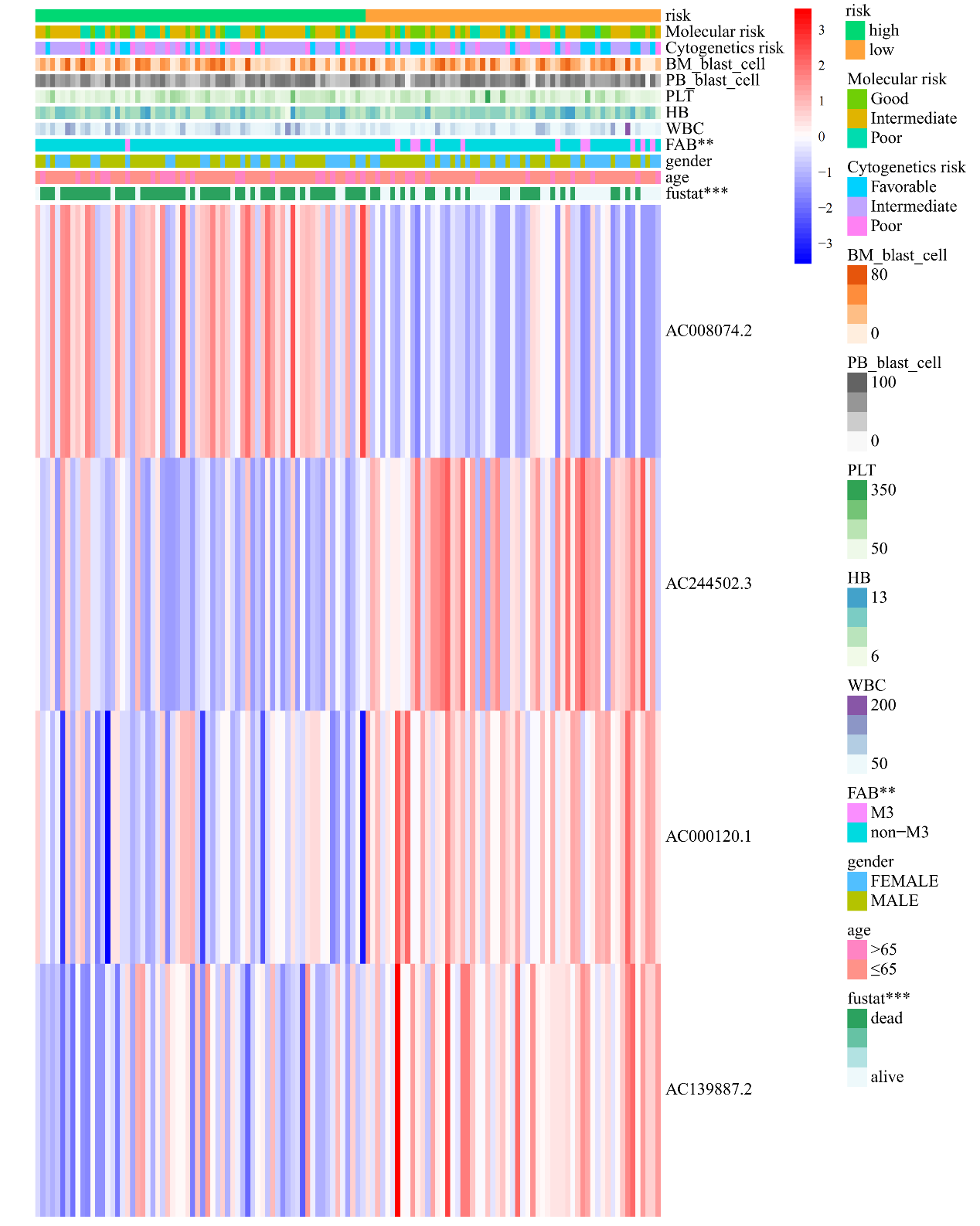


**Supplementary Figure 3 Distribution heat map of four prognostic PRlncRNA and clinicopathological variables in the high- and low-risk groups.** (Wilcoxon test, *p < 0.05; **p < 0.01; ***p < 0.001).


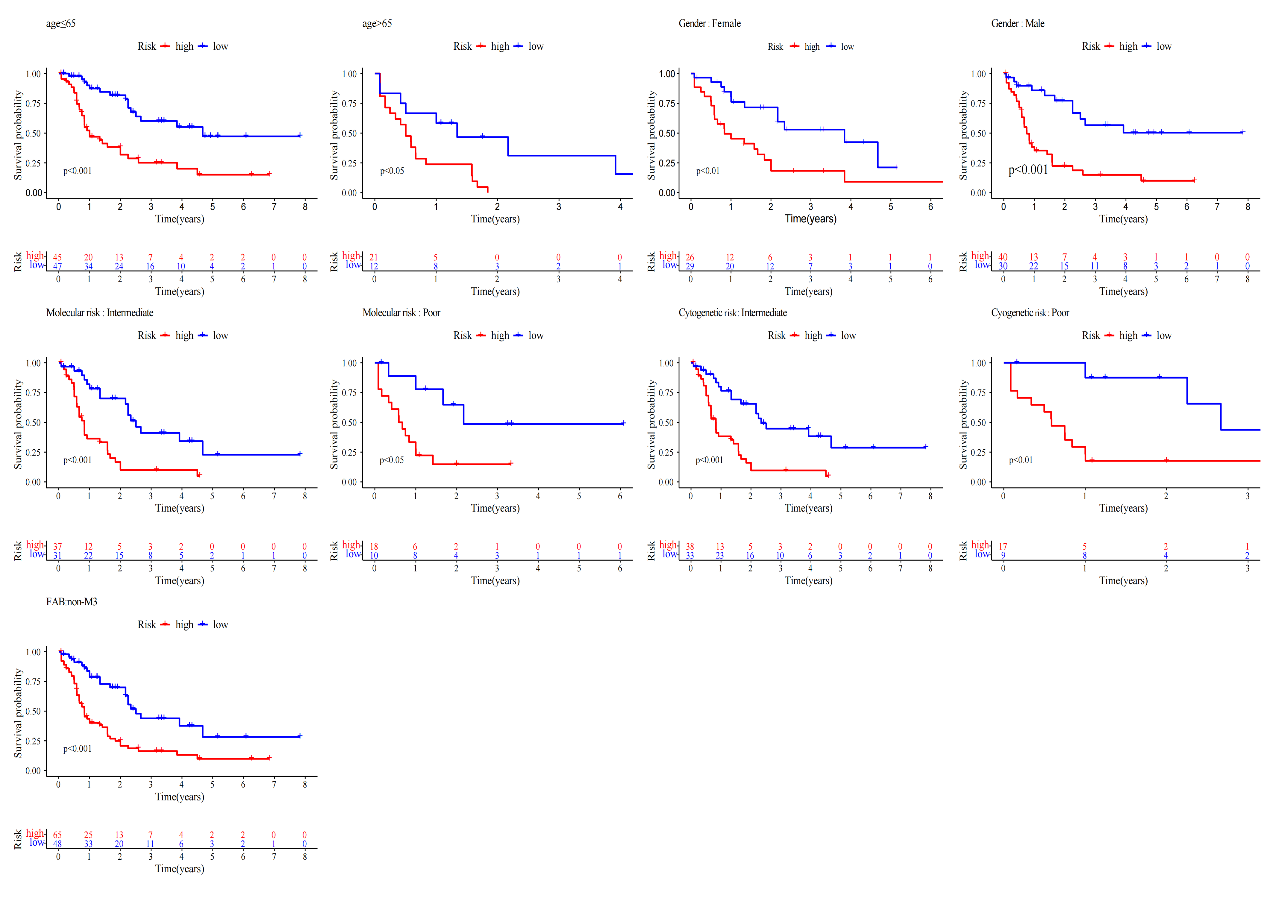


**Supplementary Figure 4** **Kaplan-Meier survival curves of high- and low-risk groups sorted according to different clinicopathological variables.**


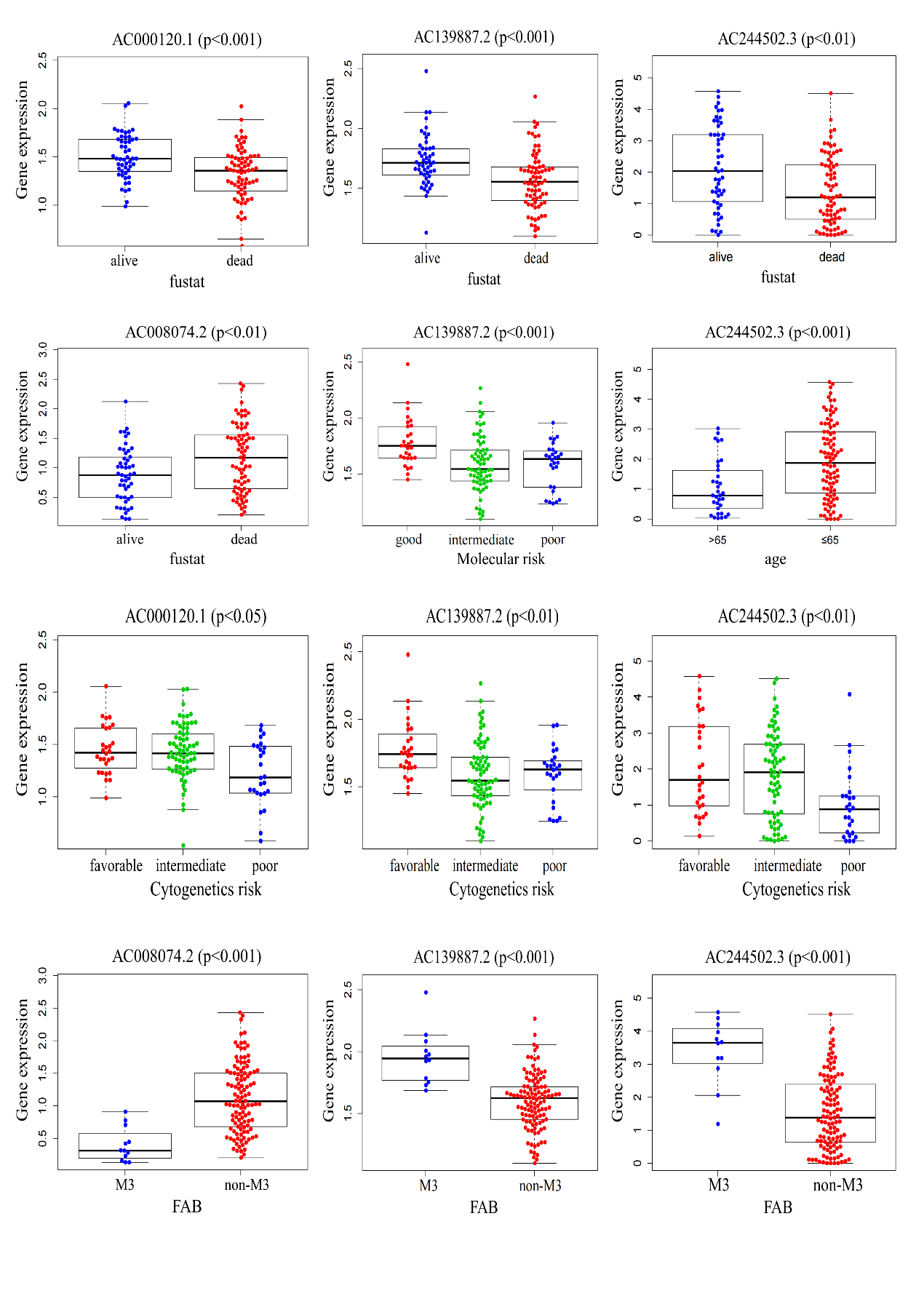


**Supplementary Figure 5 The expression of four prognostic PRlncRNA in clinicopathological variables.**


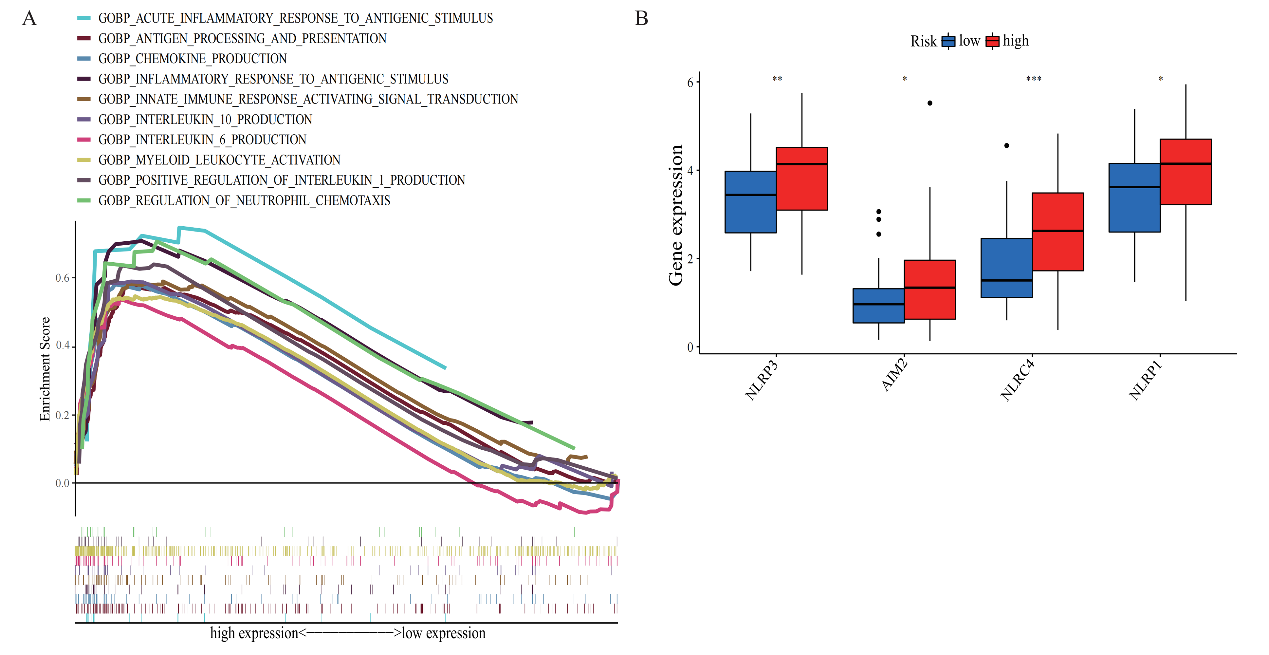


**Supplementary Figure 6** **The functional enrichment analysis of PRGs.** (A) The high-risk group enriched gene sets of GSEA-based GO analysis. (B) The expressed level of inflammasomes-related genes between two risk groups. (Wilcoxon test, *p < 0.05; **p < 0.01; ***p < 0.001).
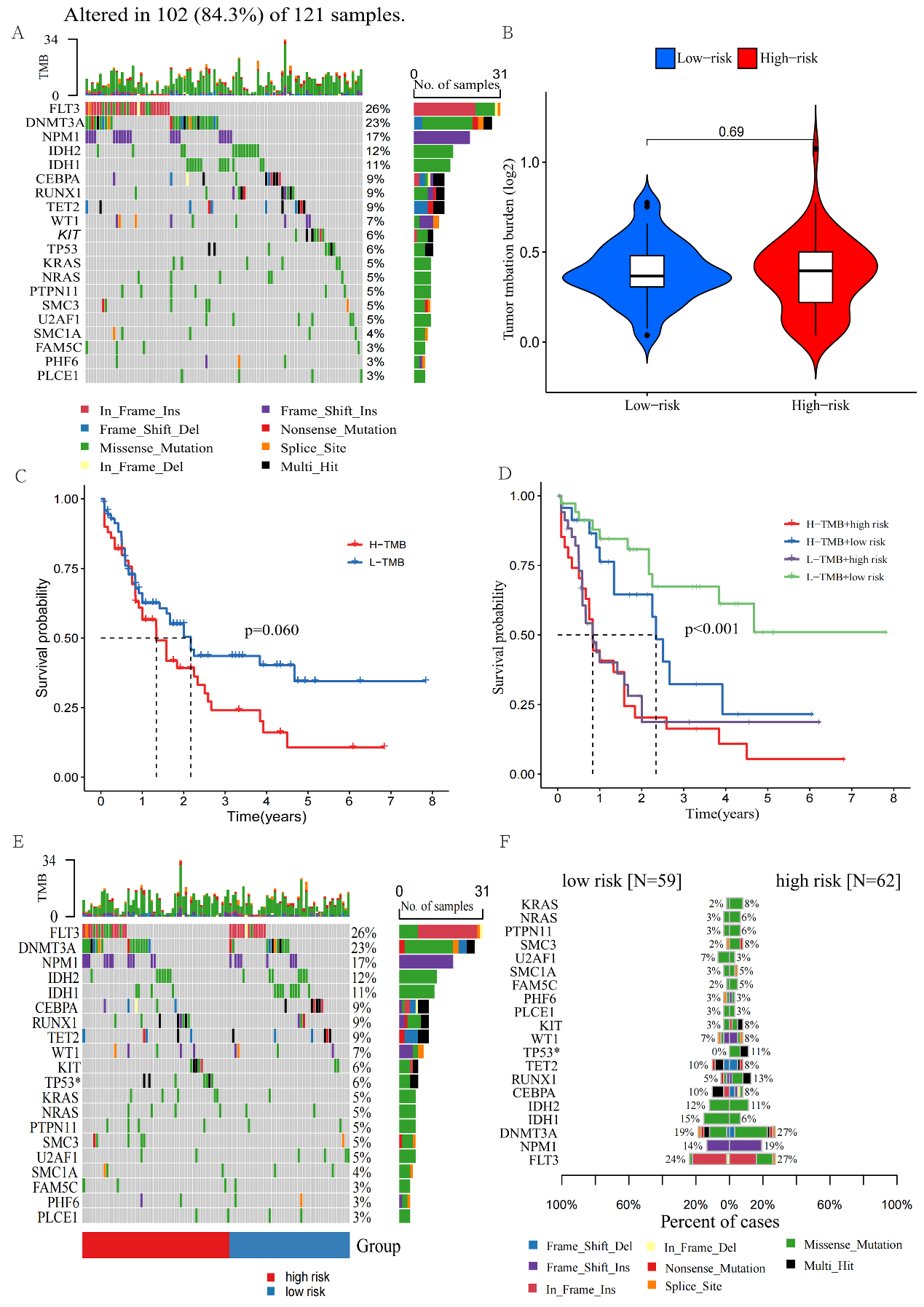


**Supplementary Figure 7 Tumor mutational burden (TMB).** (A) Waterfall of the top 20 mutated genes in the TCGA AML cohort. (B) TMB in low- and high-risk groups. (C-D) Overall survival curves stratified by the low- and high-TMB and low- and high-risk. (E-F) Waterfall of the top 20 mutated genes in two risk groups. (Wilcoxon test, *p < 0.05; **p < 0.01; ***p < 0.001).

**Supplementary Table 1** Pyroptosis-related genes.

| Protein-coding genes | | | | |
| --- | --- | --- | --- | --- |
| GSDMD | TRIM24 | GJA1 | EEF2K | ICAM1 |
| CASP1 | HMGB1 | SNIP1 | FPR2 | STK4 |
| CASP4 | NAIP | PRDM1 | HTRA1 | GSK3B |
| GSDMB | MEFV | VDR | PAK2 | PTGS2 |
| GSDMC | DHX9 | BRD4 | NDUFA13 | MST1 |
| NLRP1 | NLRP9 | AGER | CD274 | PRF1 |
| IL1B | STAT3 | IKBKE | SETD7 | PRMT5 |
| GZMB | SIRT1 | PKM | FGF21 | ELAVL1 |
| GSDMA | CTSG | IFI16 | MMP1 | MPEG1 |
| AIM2 | NEK7 | CRTAC1 | IKZF1 | STING1 |
| CASP8 | TREM2 | SEZ6L2 | BSG | ABL1 |
| GZMA | FOXO3 | SMIM1 | CEBPB | EPHA2 |
| PYCARD | TP53 | TET2 | TFAM | HDAC6 |
| CASP5 | NFE2L2 | CTSV | TRIM21 | TLR3 |
| IL18 | TXNIP | MLKL | NLRX1 | SQSTM1 |
| ZBP1 | DDX3X | PTPN11 | USF2 | CDK9 |
| CASP3 | SESN2 | MAPK14 | IL32 | IRF3 |
| NLRC4 | ELANE | APOE | SLC30A7 | UCP1 |
| CASP6 | GBP1 | ANXA1 | MALT1 | TREM1 |
| ZDHHC1 | UBR2 | SDHB | TLR2 | TSLP |
| HNP1 | ADORA3 | CHMP2A | CYCS | IL6 |
| PTEN | OSM | CHMP2B | IL1A | NLRP2 |
| DRD2 | PECAM1 | CHMP3 | IRF1 | NLRP6 |
| ADORA1 | METTL14 | CHMP4A | IRF2 | NLRP7 |
| ADORA2B | TRIM31 | CHMP4B | TP63 | NOD2 |
| ADORA2A | BAK1 | CHMP4C | CASP9 | PJVK |
| PRKACA | BAX | CHMP7 | GPX4 | PLCG1 |
| TIRAP | SCAF11 |  |  |  |
| Non-protein-coding genes | | | | |
| MIR223 | MIR135B | ZFAS1 | MIR124-1 | MIR9-1 |
| MALAT1 | MIR556 | MIR527 | MIR200C | MIR455 |
| GAS5 | MIR302A | MIR185 | MIR195 | MIR9-2 |
| MIR30C1 | MIR214 | DLX6-AS1 | MIR485 | MIR9-3 |
| MIR22 | MIR20B | MIR23A | MIR204 | MIR497 |
| NEAT1 | MIR448 | MIR520C | CDKN2B-AS1 | LINC-ROR |
| MIR125A | MIR155HG | KLF3-AS1 | HOTTIP | MIR25 |
| MEG3 | MIR155 | MIR21 | MIR141 |  |

**Supplementary Table 2** The primer sequences of signatures.

|  | Forward Primers | Reverse Primers |
| --- | --- | --- |
| AC000120.1 | 5’-ACTGCGCGTTTGTGAGCTG-3' | 5’-CGGGGAGATTCCGGTACGA-3' |
| AC008074.2 | 5’-AGTTAAAGGCAGGGCTGTTG-3' | 5’-TGAATGCTGGAATTTGTAAAGGTT-3' |
| AC139887.2 | 5’-TTTGAAGACACCACCTCGTGAT-3' | 5’-TCACCACCCAAATACATCAGCA-3' |
| AC244502.3 | 5’-AGCATCAACATTTGCAGGGC-3' | 5’-CCACGGCACAGGATCTAACA-3' |
| GAPDH | 5’-GTCTCCTCTGACTTCAACAGCG-3' | 5’-ACCACCCTGTTGCTGTAGCCAA-3' |

**Supplementary Table 3** Functional annotation for pyroptosis-related DEGs (KEGG).

| ID | Description | pvalue | p.adjust | qvalue |
| --- | --- | --- | --- | --- |
| hsa04621 | NOD-like receptor signaling pathway | 9.64E-11 | 1.80E-08 | 1.25E-08 |
| hsa05134 | Legionellosis | 4.33E-07 | 3.89E-05 | 2.69E-05 |
| hsa05130 | Pathogenic Escherichia coli infection | 6.23E-07 | 3.89E-05 | 2.69E-05 |
| hsa05132 | Salmonella infection | 4.38E-06 | 0.000204773 | 0.000141779 |
| hsa04115 | p53 signaling pathway | 3.62E-05 | 0.001353927 | 0.000937422 |
| hsa05162 | Measles | 7.80E-05 | 0.00242977 | 0.001682306 |
| hsa05417 | Lipid and atherosclerosis | 0.00011226 | 0.002998957 | 0.002076396 |
| hsa05161 | Hepatitis B | 0.000181472 | 0.004241909 | 0.002936982 |
| hsa05213 | Endometrial cancer | 0.000227392 | 0.004508222 | 0.003121369 |
| hsa05416 | Viral myocarditis | 0.000259372 | 0.004508222 | 0.003121369 |

**Supplementary Table 4** Functional annotation for pyroptosis-related DEGs (GO)

| ID | Description | pvalue | p.adjust | qvalue |
| --- | --- | --- | --- | --- |
| GO:0070269 | pyroptosis | 6.44E-16 | 1.64E-12 | 9.06E-13 |
| GO:2000116 | regulation of cysteine-type endopeptidase activity | 1.25E-13 | 1.60E-10 | 8.81E-11 |
| GO:0050727 | regulation of inflammatory response | 4.01E-12 | 3.41E-09 | 1.88E-09 |
| GO:2001056 | positive regulation of cysteine-type endopeptidase activity | 1.51E-11 | 9.64E-09 | 5.32E-09 |
| GO:0043281 | regulation of cysteine-type endopeptidase activity involved in apoptotic process | 2.01E-11 | 1.02E-08 | 5.65E-09 |
| GO:0010950 | positive regulation of endopeptidase activity | 9.99E-11 | 4.25E-08 | 2.34E-08 |
| GO:0010952 | positive regulation of peptidase activity | 2.56E-10 | 8.42E-08 | 4.65E-08 |
| GO:0052548 | regulation of endopeptidase activity | 2.64E-10 | 8.42E-08 | 4.65E-08 |
| GO:0052547 | regulation of peptidase activity | 5.85E-10 | 1.59E-07 | 8.76E-08 |
| GO:0045862 | positive regulation of proteolysis | 6.45E-10 | 1.59E-07 | 8.76E-08 |

**Supplementary Table 5** The high-risk group enriched gene sets of GSEA-based KEGG.

| Gene Set | ES | NES | NOM p-value | FDR q-value |
| --- | --- | --- | --- | --- |
| KEGG_ANTIGEN_PROCESSING_AND_PRESENTATION | 5.30E-01 | 1.69E+00 | 0.012 | 0.078 |
| KEGG_APOPTOSIS | 4.60E-01 | 1.61E+00 | 0.022 | 0.111 |
| KEGG_CHEMOKINE_SIGNALING_PATHWAY | 5.00E-01 | 1.69E+00 | 0.02 | 0.079 |
| KEGG_CYTOKINE_CYTOKINE_RECEPTOR_INTERACTION | 4.30E-01 | 1.51E+00 | 0.045 | 0.14 |
| KEGG_FC_GAMMA_R_MEDIATED_PHAGOCYTOSIS | 6.40E-01 | 1.99E+00 | 0 | 0.027 |
| KEGG_LEUKOCYTE_TRANSENDOTHELIAL_MIGRATION | 4.30E-01 | 1.58E+00 | 0.019 | 0.102 |
| KEGG_MAPK_SIGNALING_PATHWAY | 4.00E-01 | 1.62E+00 | 0.002 | 0.108 |
| KEGG_TOLL_LIKE_RECEPTOR_SIGNALING_PATHWAY | 5.30E-01 | 1.69E+00 | 0.018 | 0.083 |
| KEGG_VEGF_SIGNALING_PATHWAY | 4.10E-01 | 1.49E+00 | 0.028 | 0.153 |
| KEGG_WNT_SIGNALING_PATHWAY | 0.39 | 1.5 | 0.024 | 0.147 |

**Supplementary Table 6** The high-risk group enriched gene sets of GSEA-based Hallmark.

| Gene Set | ES | NES | NOM p-value | FDR q-value |
| --- | --- | --- | --- | --- |
| HALLMARK_APOPTOSIS | 0.43 | 1.6 | 0.019 | 0.058 |
| HALLMARK_FATTY_ACID_METABOLISM | 0.48 | 1.65 | 0.022 | 0.048 |
| HALLMARK_IL6_JAK_STAT3_SIGNALING | 0.57 | 1.74 | 0.021 | 0.035 |
| HALLMARK_INFLAMMATORY_RESPONSE | 0.57 | 1.87 | 0.004 | 0.048 |
| HALLMARK_INTERFERON_GAMMA_RESPONSE | 0.59 | 1.68 | 0.046 | 0.046 |
| HALLMARK_KRAS_SIGNALING_UP | 0.42 | 1.62 | 0.016 | 0.053 |
| HALLMARK_P53_PATHWAY | 0.46 | 1.71 | 0.01 | 0.036 |
| HALLMARK_PEROXISOME | 0.43 | 1.54 | 0.02 | 0.076 |
| HALLMARK_PI3K_AKT_MTOR_SIGNALING | 0.43 | 1.67 | 0 | 0.044 |
| HALLMARK_TNFA_SIGNALING_VIA_NFKB | 0.59 | 1.83 | 0.004 | 0.027 |

**Supplementary Table** 7 The high-risk group enriched gene sets of GSEA-based GO.

| Gene Set | ES | NES | NOM p-value | FDR q-value |
| --- | --- | --- | --- | --- |
| GOBP_ACUTE_INFLAMMATORY_RESPONSE_TO_ANTIGENIC_STIMULUS | 0.73 | 1.84 | 0.004 | 0.052 |
| GOBP_ANTIGEN_PROCESSING_AND_PRESENTATION | 0.58 | 2.02 | 0.002 | 0.03 |
| GOBP_CHEMOKINE_PRODUCTION | 0.58 | 1.91 | 0 | 0.043 |
| GOBP_INFLAMMATORY_RESPONSE_TO_ANTIGENIC_STIMULUS | 0.69 | 2.07 | 0 | 0.066 |
| GOBP_INNATE_IMMUNE_RESPONSE_ACTIVATING_SIGNAL_TRANSDUCTION | 0.58 | 1.95 | 0.004 | 0.038 |
| GOBP_INTERLEUKIN_10_PRODUCTION | 0.58 | 1.82 | 0.006 | 0.057 |
| GOBP_INTERLEUKIN_6_PRODUCTION | 0.53 | 1.85 | 0.004 | 0.052 |
| GOBP_MYELOID_LEUKOCYTE_ACTIVATION | 0.53 | 1.87 | 0.01 | 0.054 |
| GOBP_POSITIVE_REGULATION_OF_INTERLEUKIN_1_PRODUCTION | 0.64 | 1.84 | 0.004 | 0.052 |
| GOBP_REGULATION_OF_NEUTROPHIL_CHEMOTAXIS | 0.69 | 1.93 | 0 | 0.04 |
